# 4-cholesten-3-one suppresses lung adenocarcinoma metastasis by regulating translocation of HMGB1, HIF1*α* and Caveolin-1

**DOI:** 10.1038/cddis.2016.281

**Published:** 2016-09-22

**Authors:** Jinben Ma, Guobin Fu, Jing Wu, Shaoxian Han, Lishan Zhang, Ming Yang, Yong Yu, Mengyuan Zhang, Yanliang Lin, Yibing Wang

**Affiliations:** 1 Department of Anesthesiology, Shandong provincial Hospital Affiliated to Shandong University, Jinan 250021, China; 2 Department of Oncology, Shandong provincial Hospital Affiliated to Shandong University, Jinan 250021, China; 3 Department of thoracic surgery, Shandong chest Hospital, Jinan 250021, China; 4 Department of Hand and Foot Surgery, Shandong provincial Hospital Affiliated to Shandong University, Jinan 250021, China; 5 Department of Ultrasound, Shandong provincial Hospital Affiliated to Shandong University, Jinan 250021, China; 6 Department of Center Laboratory, Shandong provincial Hospital Affiliated to Shandong University, Jinan 250021, China; 7 Department of burn and plastic surgery, Shandong provincial Hospital Affiliated to Shandong University, Jinan 250021, China

## Abstract

Metastasis is a great challenge in lung adenocarcinoma (ADC) therapy. Cholesterol has been implicated in ADC metastasis. 4-cholesten-3-one, as cholesterol metabolite and analog, can substitute membrane cholesterol and increase membrane fluidity. In this study, we explored the possibility that 4-cholesten-3-one inhibited ADC metastasis. Low-dose 4-cholesten-3-one significantly restrained ADC cells migration and invasion with little effects on cells viabilities. Further investigation showed that 4-cholesten-3-one promoted ROS generation, which transiently activated AMPK*α*1, increased HIF1*α* expression, reduced Bcl-2 expression and caused autophagy. AMPK*α*1 knockdown partly suppressed 4-cholesten-3-one-induced autophagy but, neither prevented 4-cholesten-3-one-induced upregulation of HIF1*α* or downregulation of Bcl-2. 4-cholesten-3-one-induced autophagy facilitated the release of HMGB1 from nuclei to cytoplasm, blocking nuclear translocation of HIF1*α* and activation of MMP2 and MMP9. Also, 4-cholesten-3-one induced time-dependent phosphorylation of caveolin-1, Akt and NF-*κ*B. With increasing treatment time, 4-cholesten-3-one accelerated caveolin-1 internalization, but reduced the phosphorylation of Akt and NF-*κ*B, and inhibited the expression of snail and twist. These data suggested that 4-cholesten-3-one could be a potential candidate for anti-metastasis of lung adenocarcinoma.

Lung cancer is the leading cause of cancer-related death globally, with an estimated incidence of 1.3 million new cases every year.^1^ Lung adenocarcinoma (ADC) is the main common subtype of lung cancer. The high mortality of ADC is mainly attributed to early metastasis.^2^ Because metastasis is a complex and multistep process, the molecular mechanisms of ADC metastasis remain largely unknown.

Malignant cells migration is an early event of metastasis, followed by intravasation, survival in the circulation, extravasation and colonization at distant target organs.^3^ Recent studies have found the connection between cholesterol metabolism and metastases.^4, 5, 6^ Diet-induced hypercholesterolemia accelerates prostate cancer metastases to lymph node, lung and bones.^7^ Inhibition of cholesterol biosynthesis hampers the metastases of colon carcinoma and pancreatic ADC.^8, 9^ The cholesterol metabolite 27-hydroxycholesterol promotes breast cancer metastasis by activating liver X receptor.^10^ Cholesterol as an essential component of lipid rafts impacts diverse signaling molecules that mediate multiple biological functions, such as cell survival and death.^11^ A series of evidences have confirmed that the depletion of membrane cholesterol disrupts lipid rafts, resulting in cell apoptosis.^12, 13, 14^ Elevated cholesterol level has been found in various tumors, including prostate, lung, acute myeloid leukemia and breast cancer,^15, 16, 17, 18^ especially in chemoresistant tumors.^19, 20^ Cholesterol accumulation in solid tumors promotes the proliferation, differentiation and migration of tumor cells by mediating cellular surface molecules, such as caveolin-1 translocation.^21^ Depletion of membrane cholesterol suppresses the phosphorylation of Akt and ERK.^22, 23^ Furthermore, membrane cholesterol depletion also restrains the expression of BCL-2 family members.^24^ Thus, dysregulated cholesterol metabolism is likely to be implicated in tumor metastases through related signaling pathway.

Our previous results suggest that cholesterol oxidation by cholesterol oxidase from *Bordetella* species (COD-B) promotes the irreversible apoptosis of ADC cells.^25^ COD-B as a microbial flavoprotein can oxidize cholesterol to 4-cholesten-3-one. In this study, we further investigated whether 4-cholesten-3-one influenced ADC metastasis. We evidenced that low-dose 4-cholesten-3-one inhibited ADC migration *in vitro* and metastasis *in vivo* by inducing the translocations of HMGB1, HIF1*α* and caveolin-1. Our data demonstrated that translocations of HMGB1 and HIF1*α* had key roles in ADC metastasis.

## Results

### Low-dose 4-cholesten-3-one inhibited ADC cells migration and invasion with little effects on cell proliferation

**Figure 1:**
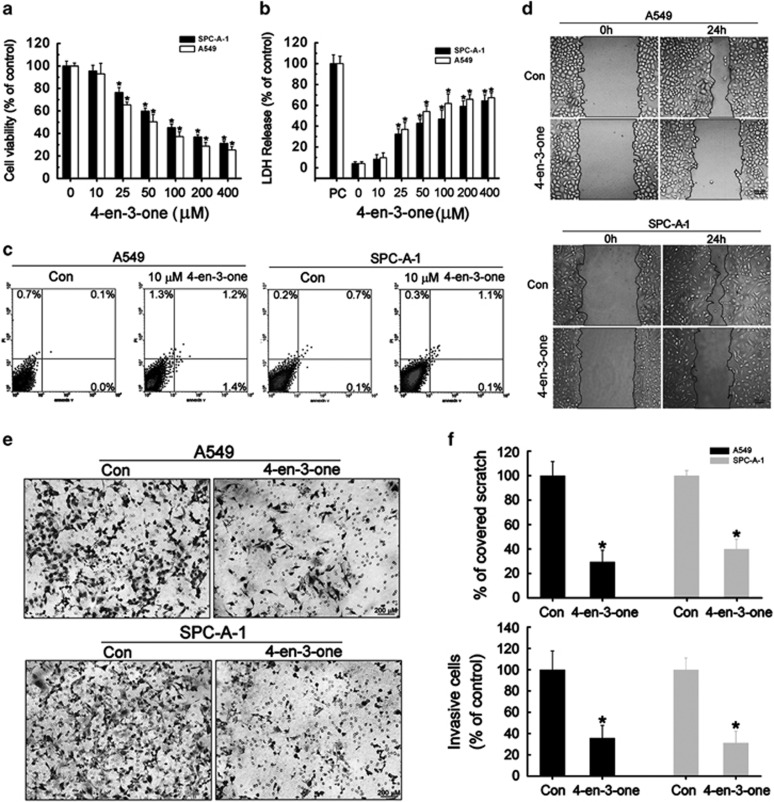
Low-dose 4-cholesten-3-one (4-en-3-one) inhibited ADC cells migration and invasion with no effects on their viabilities. (**a**) A549 and SPC-A-1 cells were serum-starved for 4 h and treated with different concentrations of 4-en-3-one for 24 h. Cell viabilities were measured by CCK8 assay. (**b**) A549 and SPC-A-1 cells were serum-starved for 4 h and treated with different concentrations of 4-en-3-one for 24 h. Cytotoxicities were measured by LDH release assay. PC represented for positive control, total cell lysate. (**c**) A549 and SPC-A-1 cells were serum-starved for 4 h and treated with 10 μM 4-en-3-one for 24 h. Cell apoptosis was determined by flow cytometry. (**d**) A549 and SPC-A-1 cells were serum-starved for 4 h and treated with 10 *μ*M 4-en-3-one for 24 h. Cell migration capacity was determined by scratch assay. (**e**) A549 and SPC-A-1 cells were serum-starved for 4 h and treated with 10 *μ*M 4-en-3-one for 24 h. Cell invasion capacity was determined by transwell assay. (**f**) Treatment with 10 *μ*M 4-en-3-one significantly inhibited ADC cells migration and invasion. Data represent means±S.D. of at least three independent experiments.*P<0.05 vs control

Our previous report has suggested that COD-B could oxidize the membrane cholesterol of ADC cells to 4-cholesten-3-one (4-en-3-one).^25^ 4-en-3-one has been demonstrated to inhibit the migration of human fibroblasts by substituting membrane cholesterol.^26^ However, it was neglected that 4-en-3-one probably inhibited cell migration by restraining cell proliferation. Moreover, it was necessary to clarify the mechanism by which 4-en-3-one conducted cell migration and invasion. To exclude the probability that 4-en-3-one inhibited cell migration and invasion via suppressing cell proliferation, we first examined the effect of 4-en-3-one on ADC cell viabilities using CCK8 assay. As shown in Figure 1a, 4-en-3-one treatment for 24 h reduced cell viabilities in a dose-dependent manner. 4-en-3-one did not depress viabilities of A549 and SPC-A-1 cells at the concentration of 10 *μ*M, but strinkingly inhibited cells growth at the concentration of 25 *μ*M up to 400 *μ*M, which were further confirmed by LDH release assay (Figure 1b). Consistently, flow cytometer analysis showed that treatment of ADC cells with 10 *μ*M 4-en-3-one did not induce cell apoptosis and necrosis (Figure 1c). Interestingly, 10 *μ*M 4-en-3-one treatment exerted a notable inhibition of ADC cells migration and invasion despite ignorable effects on cell viabilities (Figures 1d and e). Taken together, 10 *μ*M 4-en-3-one suppressed ADC cells migration and invasion with little effects on cells growth. Unless otherwise specified, 4-en-3-one was used at the concentration of 10 *μ*M in the subsequent experiments.

### Low-dose 4-en-3-one conducted signal transduction by increasing ROS level, but not through cholesterol pathway

**Figure 2:**
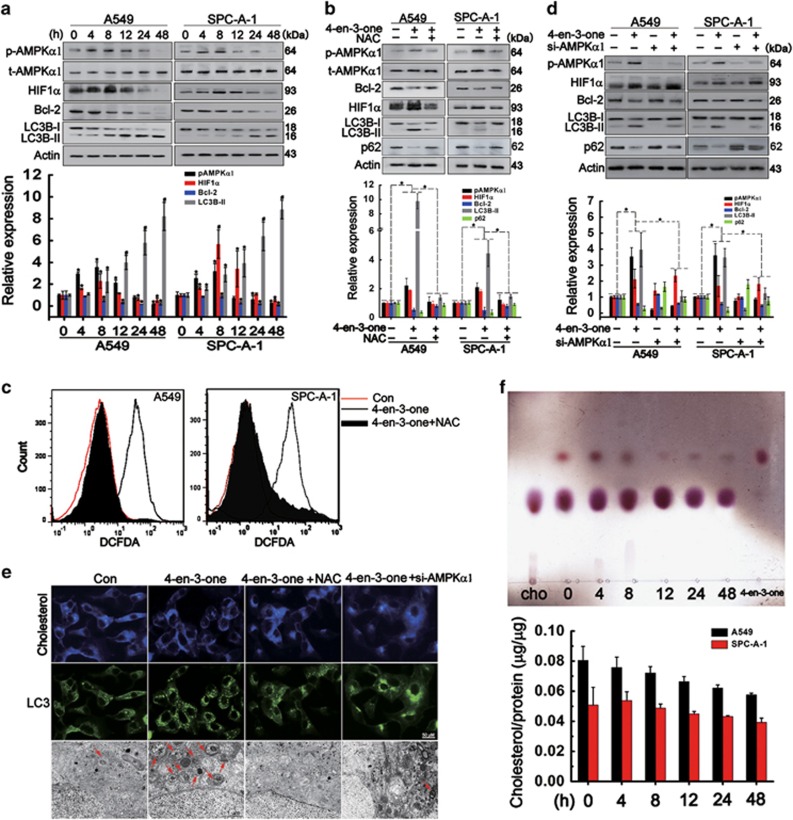
Low-dose 4-en-3-one-mediated the related signaling responses by inducing ROS generation, but not by displacing membrane cholesterol. (**a**) ADC cells were starved for 4 h and treated with 10 *μ*M 4-en-3-one for the indicated time. Western blot was used to analyze the protein levels of phosphorylated AMPK*α*1, total AMPK*α*1, HIF1*α*, Bcl-2 and LC3B. (**b**) ADC cells were pretreated with 10 mM NAC for 1 h in serum-free medium, starved for additional 3 h, and then incubated with 10 *μ*M 4-en-3-one for 8 h. Western blot was used to analyze the protein levels of phosphorylated AMPK*α*1, total AMPK*α*1, HIF1*α*, Bcl-2 and LC3B. (**c**) ROS generation was detected using H2DCF-DA by flow cytometry analysis. (**d**) ADC cells were transfected with siRNA against AMPK*α*1, starved for 4 h, and then treated with 10 *μ*M 4-en-3-one for 8 h. Western blot was used to analyze the protein levels of phosphorylated AMPK*α*1, HIF1*α*, Bcl-2 and LC3B. (**e**) After treatments as described above, A549 cells were stained by LC3 antibody to determine LC3 puncta formation and probed using filipin to determine cholesterol level. The autophagosomes were determined using transmission electron microscope. (**f**) After treatment with 10 *μ*M 4-en-3-one for indicated time, membrane lipids were extracted from A549 cells and determined using TLC analysis. The free cholesterol content was measured using free cholesterol Assay kits. Cho represented as cholesterol. Data represent means±S.D. of at least three independent experiments. *P<0.05 vs control; ^#^*P*<0.01 vs control

To explore the mechanism of how 4-en-3-one inhibited ADC cells migration and invasion, we further examined the effect of 4-en-3-one on signal transduction, ROS generation and cholesterol content. As illustrated in Figure 2a, 4-en-3-one treatment promoted a peak phosphorylation of AMPK*α*1 at 8 h followed by a decline to baseline at 48 h. After treatment with 4-en-3-one for 8 h, HIF1*α* level was also elevated at 8 h and gradually reduced with increasing treatment time. Meanwhile, 4-en-3-one inhibited Bcl-2 expression and promoted the accumulation of LC3-II in a time-dependent manner, suggesting that 4-en-3-one treatment might induce ROS generation and autophagy. To confirm this corollary, we tested the effect of *N*-acetyl-l-cysteine (NAC) on signaling response to 4-en-3-one. Figure 2b showed that NAC pre-treatment remarkably reversed signaling events induced by 4-en-3-one, which indicated an implication of ROS in signaling response to 4-en-3-one. Detecting ROS using the DCFDA, an oxidation-sensitive fluorescent reagent, also revealed that 4-en-3-one treatment facilitated the ROS generation, which was blocked by NAC pre-treatment (Figure 2c). Knockdown of AMPK*α*1 by siRNA (siAMPK*α*1) abolished AMPK*α*1 phosphorylation and partly reduced LC3-II accumulation induced by 4-en-3-one, whereas did not significantly alter the expression of Bcl-2 and HIF1*α* (Figure 2d). Figure 2e illustrated that, after 4-en-3-one treatment for 8 h, the level of membrane cholesterol was not significantly decreased while LC3 puncta were strikingly increased. Both NAC treatment and AMPK*α*1 knockdown inhibited 4-en-3-one-induced autophagy. We next assayed the levels of 4-en-3-one and cholesterol in cell membrane after 4-en-3-one treatment. The results showed that the exposure to 4-en-3-one for 4 h led to a maximum accumulation of membrane 4-en-3-one, but exerted little effects on membrane cholesterol, followed by continuous decrease in the level of 4-en-3-one with increasing treatment time (Figure 2f). These results indicated that 4-en-3-one treatment transiently increased the location of 4-en-3-one in cells membrane, elevated ROS level, promoted AMPK*α*1 phosphorylation and HIF1*α* expression, reduced Bcl-2 expression, and induced autophagy. 4-en-3-one-induced AMPK*α*1 phosphorylation partly promoted autophagy, but did not alter the expression of HIF1*α* and Bcl-2.

### 4-en-3-one-induced autophagy inhibited cells migration and invasion by mediating translocation of HMGB1 and HIF1*α*

**Figure 3:**
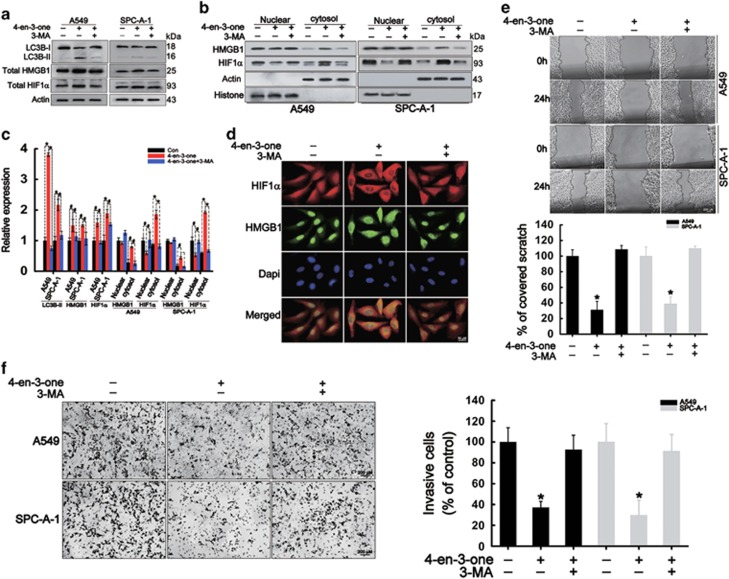
4-en-3-one inhibited ADC cells migration and invasion by regulating translocation of HIF1*α* and HMGB1. (**a** and **b**) ADC cells were pretreated with 1 mM 3-MA for 1 h in serum-free medium, starved for additional 3 h, and then incubated with 10 *μ*M 4-en-3-one for 8 h. Western blot was used to analyze the levels of LC3B, nucleic HMGB1 and HIF1*α*, cytoplasmic HMGB1 and HIF1*α*. (**c**) The relative expression of related proteins were analyzed by Scion Image software. Data are the means of triplicate independent experiments. (**d**) After treatment as described above, A549 cells were double-stained by HMGB1 and HIF1*α* antibodies to determine the translocation of HMGB1 and HIF1*α*. (**e** and **f**) ADC cells were pretreated with 1 mM 3-MA for 1 h in serum-free medium, starved for additional 3 h, and then incubated with 10 *μ*M 4-en-3-one for 24 h. ADC cells migration and invasion were determined by scratch assay and transwell assay, respectively. *P<0.01; ^#^*P*<0.05

It has been verified that autophagic stimuli promote the release of HMGB1 from nucleus to the cytosol.^27^ Our results showed that 4-en-3-one treatment reduced nucleic HMGB1 and HIF1*α*, and increased cytoplasmic HMGB1 and HIF1*α*, suggesting that 4-en-3-one promoted the release of HMGB1 from nucleus, whereas inhibited nuclear translocation of HIF1*α* (Figures 3a–c). 3-methyladenine (3-MA), an inhibitor of autophagy, not only inhibited 4-en-3-one-induced autophagy, but also abolished 4-en-3-one-induced cytoplasmic translocation of HMGB1 and facilitated nuclear translocation of HIF1*α* (Figures 3a–c). Furthermore, 3-MA suppressed expression of total HMGB1 and HIF1*α* despite presence of 4-en-3-one (Figure 3a). Immunofluorescence distinctly showed that 4-en-3-one inhibited nuclear translocation of HIF1*α* and facilitated cytoplasmic translocation of HMGB1, which was reversed by 3-MA (Figure 3d). Based on the above results, we hypothesized that 4-en-3-one-induced autophagy restrained ADC cells migration and invasion by mediating translocation of HMGB1 and HIF1*α*. As expected, 3-MA abrogated the inhibitory effects of 4-en-3-one on ADC cells migration and invasion (Figures 3e and f). In addition, AMPK*α*1 knockdown partly attenuated the inhibitory effects of 4-en-3-one on ADC cells migration (Supplementary Fig. S1). All these data suggested that 4-en-3-one-induced autophagy inhibited ADC cells migration and invasion by inducing cytoplasmic translocation of HMGB1 and blocking nuclear translocation of HIF1*α*.

### HMGB1 release blocked nuclear translocation of HIF1*α*

**Figure 4:**
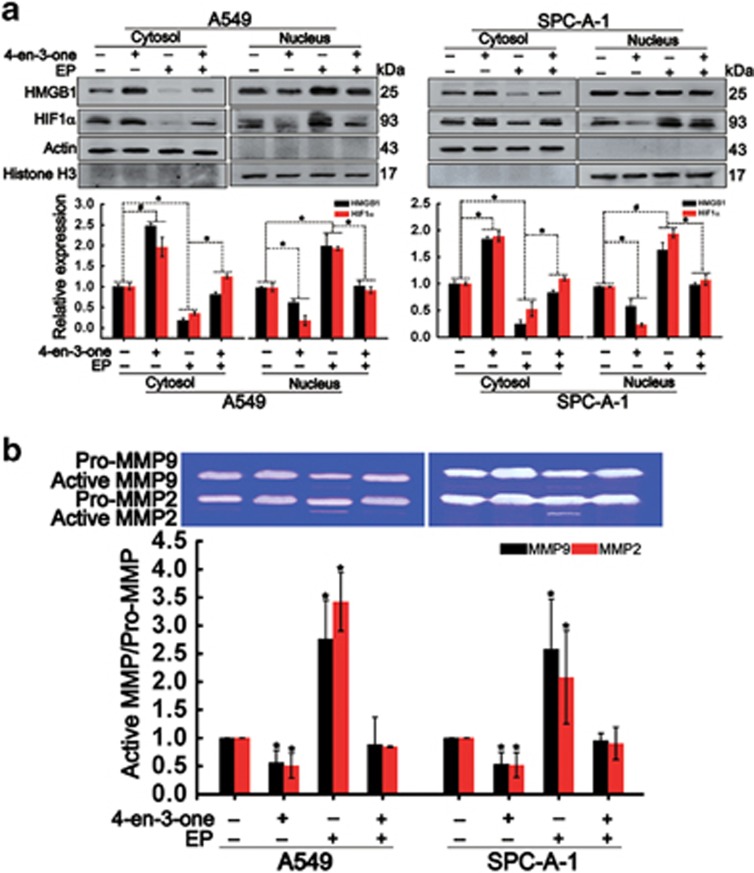
4-en-3-one blocked nuclear translocation of HIF1*α* by increasing HMGB1 translocation from nucleus to cytoplasm. ADC cells were pretreated with 5 mM ethyl pyruvate (EP) for 2 h in serum-free medium, starved for additional 2 h, and then incubated with 10 *μ*M 4-en-3-one for 8 h. (**a**) Western blot was used to analyze the levels of nucleic HMGB1 and HIF1*α*, cytoplasmic HMGB1 and HIF1*α*; (**b**) Gelatin zymography was used to analyze the activation of MMP2 and MMP9. *P<0.05; ^#^*P*<0.01

HIF1*α*, as a widely studied unit of HIF1, trends to nuclear translocation that activates the transcription of multiple genes in response to hypoxia.^28^ Elevated HIF1*α* level has been associated with tumor metastases.^29^ HMGB1 is a highly conserved DNA-binding nuclear protein. During inflammation, cell migration and tumor metastases, HMGB1 serves as an extracellular cytokine and mediates a series of signaling molecules by binding to its membrane receptors, including RAGE, TLR4 and TLR2. However, little evidences about the relationship between HMGB1 and HIF1*α* have been found. Our data showed that 4-en-3-one treatment promoted the release of HMGB1 from nucleus whereas hampered nuclear translocation of HIF1*α* (Figure 4a). Following this finding, we wondered whether blocking the release of HMGB1 from nucleus promoted nuclear translocation of HIF1*α*. EP, as an effective inhibitor of HMGB1 translocation, notably reduced the levels of cytoplasmic HMGB1 and HIF1*α*, and elevated the levels of nuclear HMGB1 and HIF1*α* despite the presence of 4-en-3-one (Figure 4), suggesting that blocking cytoplasmic translocation of HMGB1 could contribute to nuclear translocation of HIF1*α*. EP treatment also repealed the suppressive effect of 4-en-3-one on activation of MMP9 and MMP2 (Figure 4b). These results demonstrated that HMGB1 release induced by 4-en-3-one blocked the nuclear translocation of HIF1*α*, which should be responsible for activation of MMP9 and MMP2.

### 4-en-3-one induced the phosphorylation and internalization of caveolin-1

**Figure 5:**
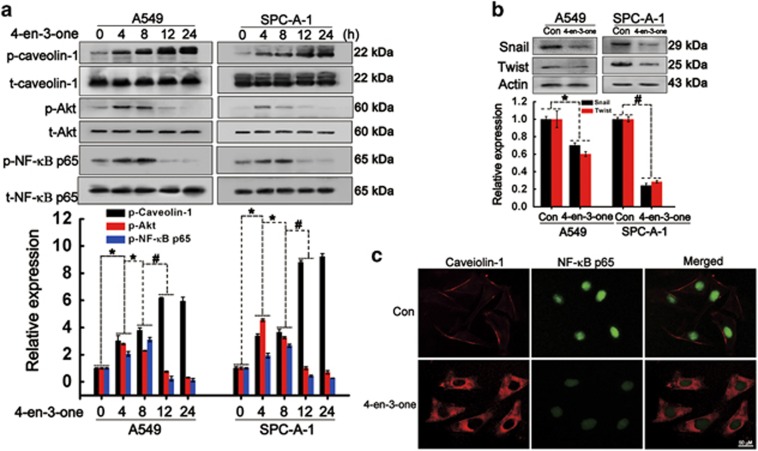
4-en-3-one induced caveolin-1 phosphorylation and internalization. (**a**) ADC cells were starved for 4 h and treated with 10 *μ*M 4-en-3-one for the indicated time. Western blot was used to analyze the phosphorylation of caveolin-1, Akt and NF-*κ*B p65. (**b**) After treatment with 10 *μ*M 4-en-3-one for 24 h, the expression of snail and twist were determined using western blot analysis. (**c**) After treatment with 10 *μ*M 4-en-3-one for 24 h, phosphorylation of caveolin-1 and NF-*κ*B p65 were analyzed by immunofluorescence. *P<0.05; ^#^*P*<0.01

Our previous report confirms that membrane cholesterol oxidation promotes the caveolin-1 internalization.^25^ We further investigated whether 4-en-3-one treatment facilitated the translocation and phosphorylation of caveolin-1. As shown in Figure 5a, 4-en-3-one promoted caveolin-1 phosphorylation as increasing treatment time. Interestingly, 4-en-3-one transiently activated Akt at 4 h, followed by gradual decline to dephosphorylation at 24 h. 4-en-3-one induced the maximum level of phosphorylated NF-*κ*B p65 at 8 h. Subsequently, 4-en-3-one distinctly reduced NF-*κ*B p65 phosphorylation at 12 and 24 h. 4-en-3-one treatment for 24 h also suppressed the expression of snail and twist that drive tumor metastases (Figure 5b). Figure 5c illustrated that 4-en-3-one treatment for 24 h remarkably propelled caveolin-1 internalization while reduced NF-*κ*B p65 phosphorylation. These results suggested that 4-en-3-one treatment contributed to the internalization and phosphorylation of caveolin-1, which should be reason for transient activation and subsequent inhibition of Akt and NF-*κ*B phosphorylation. Inhibition of NF-*κ*B phosphorylation might cause the downregulation of snail and twist after 4-en-3-one treatment for 24 h.

### 4-en-3-one induced cytoplasmic translocation of HMGB1, inhibited nuclear translocation of HIF1*α*, and reduced brain metastatic colonization of ADC cells *in vivo*

**Figure 6:**
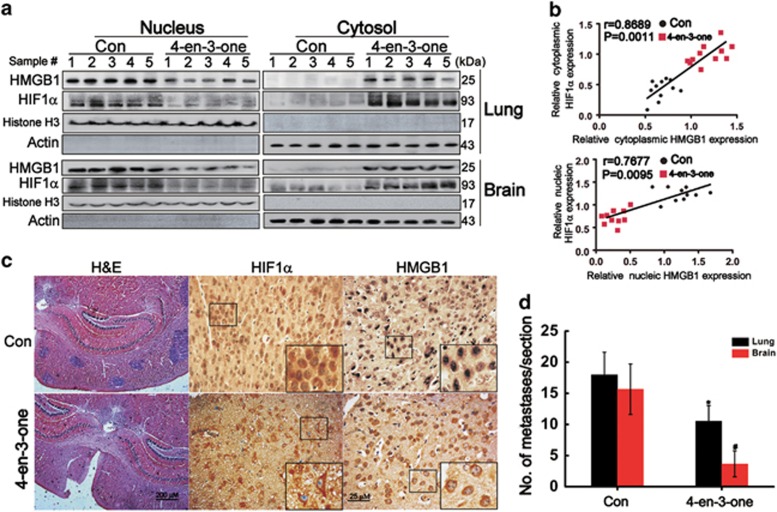
4-en-3-one induced cytoplasmic translocation of HMGB1, inhibited nuclear translocation of HIF1*α*, and reduced brain metastatic colonization of ADC *in vivo*. (**a**) After oral administration of 4-en-3-one, cells lysate of lung and brain were analyzed by western blot. Lanes 1-5 represented the lung tissues and the matched brain tissues from five mice, respectively. (**b**) The relative expression of HMGB1 and HIF1*α* in cytoplasm or nucleus was analyzed by Scion Image 4.03 software, and linear regression analysis was performed using GraphPad Prism 5.0 software. (**c**) The murine brain sections were analyzed after H&E and immunohistochemistry using antibodies against HIF1*α* and HMGB1. (**d**) Tumor colonies in lungs and brains were counted. *P<0.05; ^#^*P*<0.01

Next, we evaluated the effect of 4-en-3-one on the translocation of HMGB1 and HIF1*α in vivo*. Owing to the anti-obesity effect of 4-en-3-one,^30^ we tested the effect of 4-en-3-one on body weights of mice. The body weights of mice fed with 0.03% 4-en-3-one were similar to those of control mice (Supplementary Fig.S2). When the concentration of 4-en-3-one in diet was raised to 0.06% or more, the body weights of mice were significantly reduced compared with the control group (Supplementary Fig.S2). The diet containing 0.03% 4-en-3-one did not affect the feed consumption of mice (Supplementary Table S1). When the mice were fed with 4-en-3-one-supplemented diet (0.03%) for 2 weeks, the serum 4-en-3-one was elevated to about 5.24 *μ*M. After continuous feeding with 4-en-3-one for 9 weeks, the serum 4-ten-3-one was ~5.42 *μ*M (Supplementary Fig.S3F), whereas serum cholesterol and triglyceride were not affected (Supplementary Fig.S3D and E). Although the serum 4-en-3-one did not approach 10 *μ*M used in *in vitro* experiments, to exclude the effect of body weight loss, the concentration of 4-en-3-one was set as 0.03% in this study. Compared with the control diet, whatever in lung or brain tissues, oral administration of 4-en-3-one significantly reduced the expression of nuclear HMGB1 and HIF1*α*, and increased the levels of cytoplasmic HMGB1 and HIF1*α* (Figure 6a), suggesting that 4-en-3-one induced the translocation of HMGB1 and HIF1*α* similar to those occurred *in vitro*. The level of nuclear HMGB1 was positively correlated to the level of nuclear HIF1*α*, and the level of cytoplasmic HMGB1 was also positively correlated to the level of cytoplasmic HIF1*α* (Figure 6b). As expected, oral administration of 4-en-3-one distinctly reduced brain metastatic nodules (Figure 6c).

## Discussion

Cholesterol is an essential component of plasma membrane in eukaryotes. As a cholesterol analog, 4-cholesten-3-one (4-en-3-one) can increase membrane fluidity by displacing membrane cholesterol, leading to translocation of membrane molecules, such as CD44 shedding.^5^ Given that cholesterol functions as a membrane adhension molecule and contributes to tumor invasion and metastasis,^5^ it was hypothesized that 4-en-3-one might interfere with tumor invasion and metastasis by substituting membrane cholesterol. To exclude the possibility that 4-en-3-one suppressed tumor invasion and metastasis by reducing cells viabilities, we first investigated the effect of 4-en-3-one on ADC cells viabilities. Our results evidenced that exposure of ADC cells to 10 *μ*M 4-en-3-one for 24 h did not inhibit viabilities of A549 and SPC-A-1 cells, but significantly inhibited cells migration and invasion. Our previous report has confirmed that COD, catalyzing oxidation of cholesterol to 4-en-3-one, results in irreversible apoptosis of ADC cells.^25^ However, we ignored whether COD inhibited cells migration with no effects on cells viabilities. Maarit *et al.* demonstrate that treatment with COD or 4-en-3-one for 22 h remarkably suppresses cells migration of fibroblasts by substituting membrane cholesterol, in which their effects on cells viabilities are also neglected.^26^

Because 4-en-3-one can replace membrane cholesterol,^5, 26^ further investigation was performed to determine whether the suppressive effect of lower dose 4-en-3-one on cells migration and invasion was due to substitute it for membrane cholesterol. Our data showed that low-dose 4-en-3-one inhibited cells migration and invasion in a cholesterol-independent manner, which was different from previous report.^26^ Then, the question how 4-en-3-one functioned in ADC cells was put forward. We estimated the effect of 4-en-3-one on the several signaling responses, including AMPK*α* phosphorylation, the expression of HIF1*α* and Bcl-2, and autophagy. 4-en-3-one treatment promoted AMPK*α*1 phosphorylation and HIF1*α* expression, inhibited Bcl-2 expression, and induced autophagy in a time-dependent manner. The Bcl-2 protein is closely associated with ROS generation.^25^ NAC pre-treatment confirmed that 4-en-3-one induced ROS generation to mediate signaling responses. ROS has been implicated in autophagy by inhibiting Bcl-2 expression, which is correlated to cells migration.^31^ Interestingly, AMPK*α*1 knockdown induced a partial attenuation in autophagy induced by 4-en-3-one, whereas did not notably alter expression of Bcl-2 and HIF1*α*.

3-MA serves as an autophagy inhibitor and is widely used to explore the functions of autophagy.^32^ Our data revealed that inhibition of autophagy by 3-MA significantly blocked the cytoplasmic translocation of HMGB1, whereas promoted nuclear translocation of HIF1*α* despite the presence of 4-en-3-one. As a result, 3-MA treatment abolished the suppressive effect of 4-en-3-one on ADC cells migration and invasion. These results indicated that 4-en-3-one-induced autophagy promoted the translocation of HMGB1 from nucleus and restrained nuclear translocation of HIF1*α*, which impaired ADC cells migration and invasion. Consistent with our observation, nuclear translocation of HIF1*α* has been found to accelerate tumors metastases.^33, 34, 35, 36, 37, 38^

Under hypoxic conditions, HIF1*α* tends to translocate into nuclei and activates the transcription of genes involved in essential cancer progressions including tumor metastasis and invasiveness. However, the precise mechanism of HIF1*α* translocation remains largely unknown. Ethyl pyruvate (EP) has been identified as an efficient blocker for HMGB1 release from nuclei,^27, 39^ which was confirmed by our data. EP treatment also promoted nuclear translocation of HIF1*α*, indicating that inhibition of HMGB1 release contributed to nuclear translocation of HIF1*α*. Several evidences have verified that EP could stabilize HIF1*α* protein and increase the transcription of HIF1*α* target genes,^40, 41^ which was in line with our findings that EP enhanced activities of MMP2 and MMP9.

Owing to our previous results that cholesterol oxidation promotes caveolin-1 internalization, we investigated whether 4-en-3-one promoted caveolin-1 phosphorylation and internalization. Our results revealed that 4-en-3-one treatment induced phosphorylation of caveolin-1, Akt and NF-*κ*B p65 in a time-dependent manner. 4-en-3-one treatment also accelerated caveolin-1 internalization. Because caveolin-1 phosphorylation increases caveolae internalization, it was reasonable that 4-en-3-one stimulated phosphorylation and internalization of caveolin-1. Considering that PI3K/Akt localize on caveolae and caveolae internalization inhibits Akt phosphorylation,^12^ 4-en-3-one-induced caveolin-1 internalization possibly contributed to Akt dephosphorylation. The transient phosphorylation of Akt probably responded to ROS generation. Akt phosphorylation promotes NF-*κ*B activation,^42^ which explained our observation that Akt phosphorylation was prior to NF-*κ*B phosphorylation. 4-en-3-one treatment for 24 h significantly inhibited the expression of snail and twist that were tightly correlated to cancer cells migration. NF-*κ*B phosphorylation has an important role in transcriptions of snail and twist.^43^ Combined with the result that 4-en-3-one treatment for 24 h suppressed NF-*κ*B phosphorylation, it was likely that the inhibitory effect of 4-en-3-one on ADC cells migration was partly due to block caveolin-1/Akt/NF-*κ*B-signaling pathway.

Because the anti-obesity effect of 4-en-3-one has been observed,^30^ to exclude the effect of body weight loss on ADC metastasis *in vivo*, mice were fed with the diet containing 0.03% 4-en-3-one that did not affect body weights of mice. Although serum 4-en-3-one did not approach 10 *μ*M, a pharmacologic dose used in *in vitro* experiments, 4-en-3-one treatment still facilitated HMGB1 translocation from nuclei and restrained nuclear translocation of HIF1*α in vivo*. As expected, 4-en-3-one treatment reduced the brain metastatic colonization of ADC cells. Taken together, 4-en-3-one inhibited ADC cells migration/invasion and brain metastases mainly via regulating translocation of HMGB1 and HIF1*α*.

**Figure 7:**
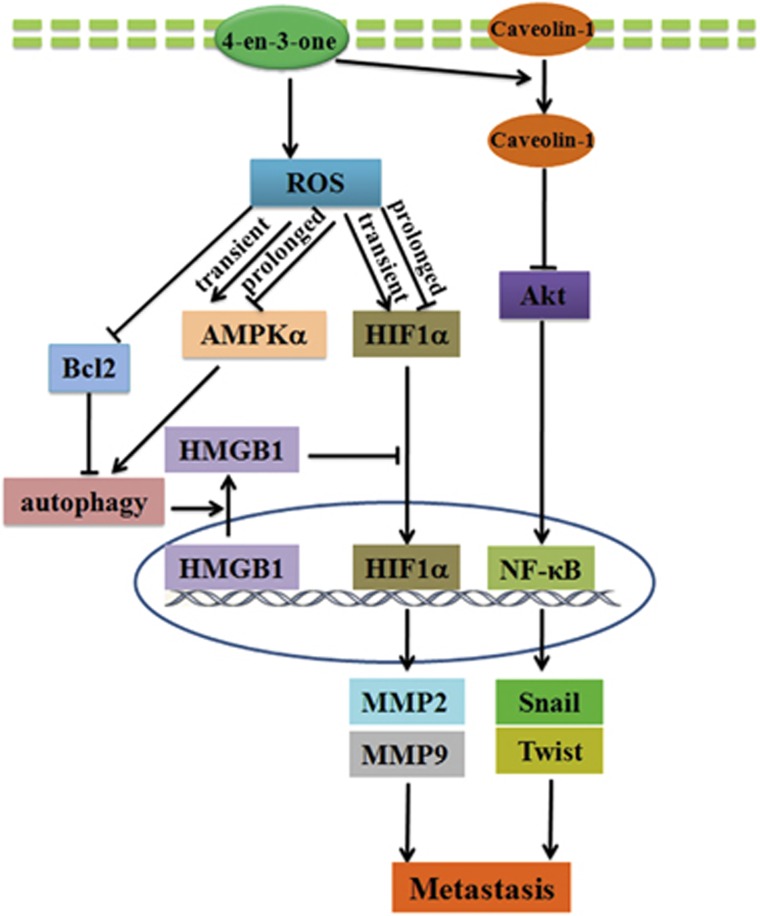
Schematic diagram illustrated the possible role of 4-en-3-one in regulating ADC migration and invasion

In summary, we evidenced that low-dose 4-en-3-one distinctly inhibited ADC cells migration and invasion with little effects on cells viabilities. Further investigation demonstrated that 4-en-3-one induced ROS generation. The elevated ROS level transiently activated AMPK*α*1 and gradually inhibited AMPK*α*1 phosphorylation with increasing treatment time, which was in paralleled with HIF1*α*. 4-en-3-one-induced ROS also decreased Bcl-2 level and promoted autophagy. AMPK*α*1 knockdown partly inhibited autophagy, whereas did not alter the expression of HIF1*α* and Bcl-2. 4-en-3-one-induced autophagy accelerated HMGB1 release from nuclei to cytoplasma, blocking nuclear translocation of HIF1*α*. Decrease in nuclear HIF1*α* level suppressed activation of MMP2 and MMP9, which was mainly responsible for the inhibitory effect of 4-en-3-one on ADC cells migration and invasion. 4-en-3-one also induced the phosphorylation of caveolin-1, Akt and NF-*κ*B in a time-dependent manner. With increasing treatment time, 4-en-3-one restrained the phosphorylation of Akt and NF-*κ*B, and facilitated caveolin-1 internalization, suppressing the expression of snail and twist, which should be another reason for the inhibitory effect of 4-en-3-one on ADC cells migration (Figure 7).

## Materials and Methods

### Antibodies and reagents

The monoclonal antibodies (mAbs) and polyclonal antibodies (pAbs) were used as follows: anti-Akt (pAb) and anti-Bcl-2 (pAb) were from Anbo Biotechnology Company (San Francisco, CA, USA); anti-phospho-Akt (Ser473, pAb), anti-HIF1*α* (pAb), anti-HMGB1 (pAb), anti-caveolin-1 (pAb), anti-AMPK*α*1 (pAb), anti-phospho-AMPK*α*1 (pAb), anti-beta-actin (pAb) and anti-Histone H3 (pAb) were purchased from Abcam (Cambridge, MA, USA); anti-LC3B (pAb), anti-phosphor-Caveolin-1(pAb), anti-NF-*κ*B p65(mAb) and anti-phosphor-NF-*κ*B p65(mAb) were purchased from Cell Signaling Technology (Beverly, MA, USA). RIPA lysis buffer was purchased from Cell Signaling Technology. The *In Situ* Cell Death Detection Kit was purchased from Roche Applied Science (Shanghai, China). Cell counting kit 8 was purchased from Dojindo Laboratories (Tokyo, Japan). 4-cholesten-3-one and cholesterol were purchased from Sigma Chemical Co. (St. Louis, MO, USA).

### Cell culture

The human lung ADC cell lines A549 and SPC-A-1 were purchased from American Type Culture Collection (Manassas, VA, USA). Cells were cultured in Dulbecco's modified Eagle's medium (Hyclone, Logan, Utah, USA) supplemented with 10% fetal bovine serum (Hyclone), 100 U/ml penicillin, 100 U/ml streptomycin and 0.03% l-glutamine at 37 ^o^C in 1% O_2_, 5% CO_2_ and 94% N_2_.

### Cell treatments

The cells were plated at 1.0 × 10^5^ in a six-well microplate. After being grown to ~80% confluence, the cells were serum-starved for 4 h using 0.1% BSA in medium before treatments. 4-cholesten-3-one was dissolved in ethanol to prepare 100 mM stock solution. The cells were exposed to the indicated concentrations of 4-cholesten-3-one for 24 h.

### Cell viability assay

The cells were seeded at 1 × 10^4^ in 100 *μ*l culture medium in a 96-well microplate. After treatments, 10 *μ*l WST-8 dye was added to each well and further incubated for 2 h at 37 ^o^C. The absorbance was measured at 450 nm using SpectraMax M2.

### LDH release assay

The cells were seeded at 2 × 10^5^ in 100 *μ*l culture medium in a 96-well microplate. After treatments, cytotoxicity was determined by measuring LDH release using LDH Release Assay Kit (Thermo, Pittsburgh, PA, USA). In brief, LDH Release Assay KitLDH Release Assay KitLDH Release Assay Kitsupernatants were collected by centrifugation at 400 × *g* for 5 min. In total, 50 *μ*l of supernatant from each well was transferred to a new 96-well microplate and mixed with 50 *μ*l reaction mixture in dark at room temperature for 30 min. After adding 50 *μ*l stop solution, the absorbance was detected at 490 and 680 nm using a SpectraMax M2, which was used to calculate LDH activity. Total cell lysate were set as 100%.

### Cholesterol assay

The treated cells were fixed with 4% paraformaldehyde for 30 min. After rinsing three times, the cells were stained with 100 *μ*g/ml filipin for 1 h. The images were acquired using Nikon fluorescence microscope.

After treatment, A549 cell membranes were prepared as previously described.^25^ In brief, A549 cells were suspended into the buffer (20 mM Tris, 250 mM sucrose, 1.0 mM EDTA, 10 mM iodoacetamide, 200 *μ*M PMSF, 1 mM DTT, 10 mg/ml aprotinin, 10 mg/ml leupeptin and 0.02% sodium azide, pH 7.4), followed by sonication on ice. The membrane fractions were separated from the cytoplasmic fractions by centrifugation at 100 000 × *g* for 1 h. The membrane cholesterol was extracted with chloroform and methanol (2:1, v/v). After centrifugation, the chloroform layer was spotted onto a thin layer chromatography plate (GF_254_, 0.25 mm thickness, 10 × 10 cm). The plate was developed in dichloromethane: petroleum ether: ethyl acetate (7.7:2:0.3), and was visualized by exposing to 10% sulfuric acid.

Free cholesterol contents in ADC cells were determined using free cholesterol Assay kits (Applygen Technologies, Beijing, China) according to the manufacturer's instructions.

### Flow cytometer analysis

Flow cytometer analysis was performed to determine the cells apoptosis. Treated cells were harvested using trypsin without EDTA and phenol red. Subsequently, cells were incubated with FITC-conjugated annexin-V reagent (2.5 mg/ml) and PI (5 mg/ml), and were detected using flow cytometer analysis.

### Protein extraction

To obtain total protein, the treated cells were harvested and lyzed using RIPA lysis buffer (Thermo). Subsequently, the supernatant was collected by centrifugation at 10 000 × *g* for 20 min. The isolation of cytoplasmic and nuclear proteins was performed using a nuclear and cytoplasmic protein extraction kit (Sangon Biotech, China) according to the manufacturer's protocol. In brief, cells were bathed in pre-cooled PBS and scraped into a microcentrifuge tube. After centrifugation, the cell pellets were collected and resuspended in 1.0 ml ice-cold buffer A (adding 1 *μ*l DTT, 10 *μ*l PMSF and 1 *μ*l protease inhibitor before use), followed by vigorous vortex for 15 s and ice-bath for 15 min. Subsequently, 10 *μ*l ice-cold buffer B was supplemented, followed by vigorous vortex for 5 s and ice-bath for 1 min. After vigorous vortex for 5 s, the supernatant was collected as the cytoplasmic protein by centrifugation at 16 000 × *g* and 4 °C for 5 min. The pellet was proceeded to extract nuclear protein. The pellet was placed in 0.1 ml ice-cold buffer C (adding 0.1 *μ*l DTT, 1.0 *μ*l PMSF and 0.1 *μ*l protease inhibitor before use), followed by vigorous vortex for 15 s and ice-bath for 40 min. The supernatant was collected as the nuclear protein by centrifugation at 16 000 × *g* for 5 min. The protein concentration was determined using the bicinchoninic acid method.

### Western blot analysis

Equal amounts of proteins were separated on sodium dodecyl sulfate polyacrylamide gel electrophoresis and transferred onto polyvinylidene fluoride membranes. Membranes were blocked with 3% BSA and incubated with the primary antibodies overnight at 4 ^o^C, followed by incubation with horseradish peroxidase-conjugated secondary antibodies for 1 h at room temperature. The signals were detected using the enhanced chemiluminescence method and quantified using Scion Image 4.03 software.

### Immunofluorescence

Cells were grown on cover slips and fixed with 4% paraformaldehyde for 15 min, permeabilized with 0.2% Triton X-100 in PBS for 10 min, and blocked with 2% BSA in PBS for 1 h at room temperature. After rinsing in PBST, cells were incubated with the indicated primary antibodies overnight at 4 ^o^C. After rinsing in PBST, cells were incubated with the corresponding secondary antibody for 1 h. Subsequently, cells were counterstained with DAPI and visualized using a Nikon fluorescence microscope.

### Transmission electron microscopy

The treated cells were fixed with 2% glutaraldehyde and 2% paraformaldehyde in phosphate buffer (0.1 M, pH 7.4). Subsequently, cells were post-fixed with 1% osmium tetroxide, followed dehydration with graded alcohols. After being embedded in epoxy resin, thin sections (70 nm) were obtained, mounted on copper grids, and stained with 2% uranyl acetate and 1% lead citrate for observation by a transmission electron microscope (JEM-1200E, JEOL, Tokyo, Japan).

### Gelatin zymography

Gelatin zymography analysis was performed to determine the activities of MMP2 and MMP9 as previously described.^44^ In brief, after treatments, conditioned media were collected and diluted in Laemmli buffer without DTT under non-denaturating conditions. Equal amounts of samples were subjected to 8% SDS-PAGE containing 1 mg/ml gelatin. After electrophoresis, the gels were rinsed with 2.5% Triton X-100 and Tris-HCl (pH 7.5) before incubation in the renaturation buffer (50 mM Tris-HCl, 1 *μ*M ZnCl_2_, 5 mM CaCl_2_, 0.02% sodium azide, pH 7.6) overnight at 37 °C. Gels were stained in 0.5% Coomassie Brilliant Blue and destained in destaining buffer (30% methanol, 10% acetic acid in H_2_O). The activities of MMP2 and MMP9 were determined by quantifying the area of clear zones under the blue background.

### Migration assay

To determine the effect of 4-cholesten-3-one on cell migration, the scratch assay was performed as previously described.^45^ In brief, the cells were plated in six-well plates and cultured overnight. The formed cell monolayer was scratched using a 200 *μ*l pipette tip. After removing the debris and floating cells using fresh media, the cells underwent indicated treatments for 24 h and visualized using a Nikon fluorescence microscope.

### Invasion assay

Cell invasion capability was evaluated using transwell assay. In brief, the cells were seeded into the upper chambers with 8 *μ*m pore size membrane coated with Matrigel (BD Biosciences, Billerica, MA, USA). After treatments for 24 h, non-invading cells were removed from the upper membrane surface using cotton swabs, and cells attached to the bottom membrane surface were stained with crystal violet. The images were captured using a LEICA microscope.

### Mice xenograft models

Male 5-week-old BALB/c nude mice were purchased from Weitonglihua Animal Center (Beijing, China) and maintained under specific pathogen-free conditions in the animal facility. All procedures were approved by the Institutional Laboratory Animal Care and Use Committee at Shandong provincial hospital. For examining the effect of 4-cholesten-3-one on metastases *in vivo*, mice were fed a control diet or 4-cholesten-3-one-supplemented diet (0.03%) for 2 weeks prior to the injection of tumor cells. The A549 cells (1 × 10^6^ in 100 *μ*l PBS) were injected into the left ventricle of mice. The mice continued to be fed the corresponding diet for 7 weeks. Subsequently, the mice were killed and the lungs and brains were removed for histological analysis.

### Statistical analysis

Statistical analysis was performed using GraphPad Prism 5.0. The data were presented as the mean±S.D.s from at least three independent experiments. The differences between two groups were evaluated by Student's *t*-test. The differences among multiple groups were evaluated by one-way ANOVA. *P*<0.05 was considered to be statistically significant.

## Abbreviations

ADC: lung adenocarcinoma
COD: cholesterol oxidase
HIF1*α*: hypoxia-inducible factor 1 alpha
HMGB1: high mobility group box 1
MS: mass spectra
ROS: reactive oxygen species
LXR: liver X receptor
NAC: *N*-acetyl-l-cysteine
MMP: matrix metalloproteinase

## Supplementary Material

Supplementary Informations
