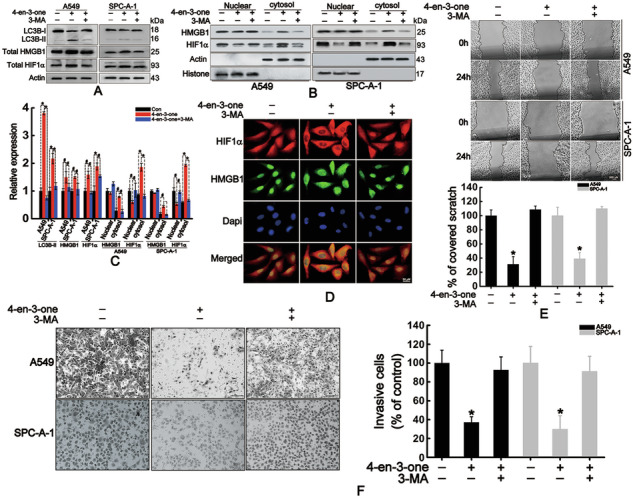# Correction: 4-cholesten-3-one suppresses lung adenocarcinoma metastasis by regulating translocation of HMGB1, HIF1α and Caveolin-1

**DOI:** 10.1038/s41419-025-08408-z

**Published:** 2026-01-28

**Authors:** Jinben Ma, Guobin Fu, Jing Wu, Shaoxian Han, Lishan Zhang, Ming Yang, Yong Yu, Mengyuan Zhang, Yanliang Lin, Yibing Wang

**Affiliations:** 1 Department of Anesthesiology, Shandong provincial Hospital Affiliated to Shandong University, Jinan, 250021 China; 2 Department of Oncology, Shandong provincial Hospital Affiliated to Shandong University, Jinan, 250021 China; 3 Department of thoracic surgery, Shandong chest Hospital, Jinan, 250021 China; 4 Department of Hand and Foot Surgery, Shandong provincial Hospital Affiliated to Shandong University, Jinan, 250021 China; 5 Department of Ultrasound, Shandong provincial Hospital Affiliated to Shandong University, Jinan, 250021 China; 6 Department of Center Laboratory, Shandong provincial Hospital Affiliated to Shandong University, Jinan, 250021 China; 7 Department of burn and plastic surgery, Shandong provincial Hospital Affiliated to Shandong University, Jinan, 250021 China

Correction to: *Cell Death and Disease* 10.1038/cddis.2016.281, published online 22 September 2016

During the review of this work, the authors identified several inadvertent errors in the figure preparation process. The original data were re-examined, confirming that these issues resulted from unintentional mistakes during the assembly of the figures or manuscript. The specific corrections are as follows:

1. An overlap is observed in the Transwell experiment images between Figure 1E and Figure 3F. A partial duplication also exists in Figure 1D.
2. The electron microscopy image shown in Figure 2E was incorrectly assembled during figure preparation.
3. Acknowledgements. This work was supported by the Promotive research fund for excellent young and middle-aged scientists of Shandong Province (Grant No. BS2011SW036; BS2013YY065), and the Science and Technology Development Plan Project of Shandong Province (Grant No. 2014GSF118157).

Although these errors do not affect the overall conclusions of the study, the authors sincerely apologize for any confusion or inconvenience they may have caused.

Original Fig. 1
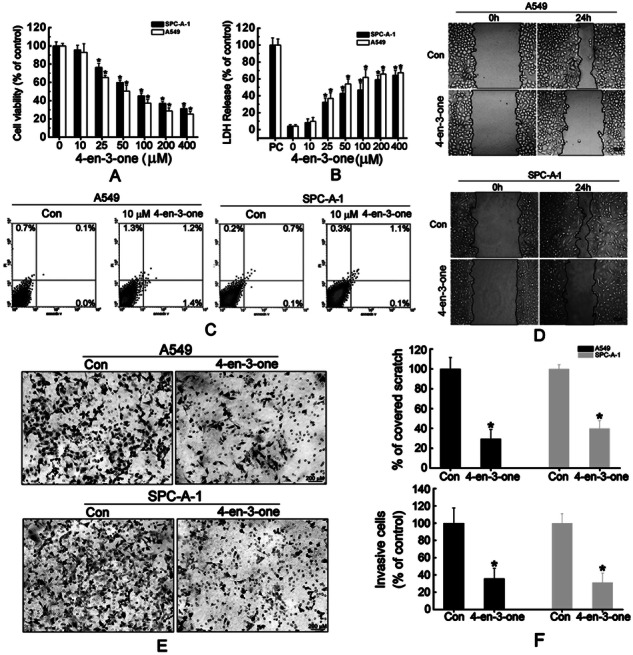


Amended Fig. 1
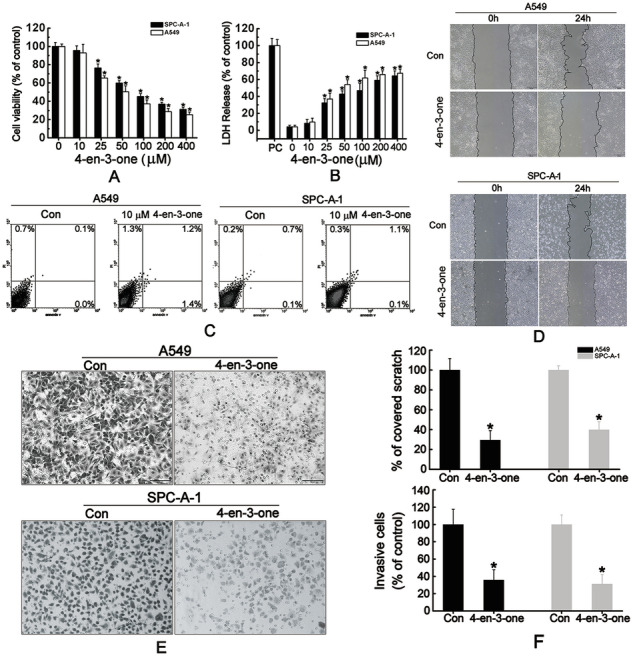


Original Fig. 2
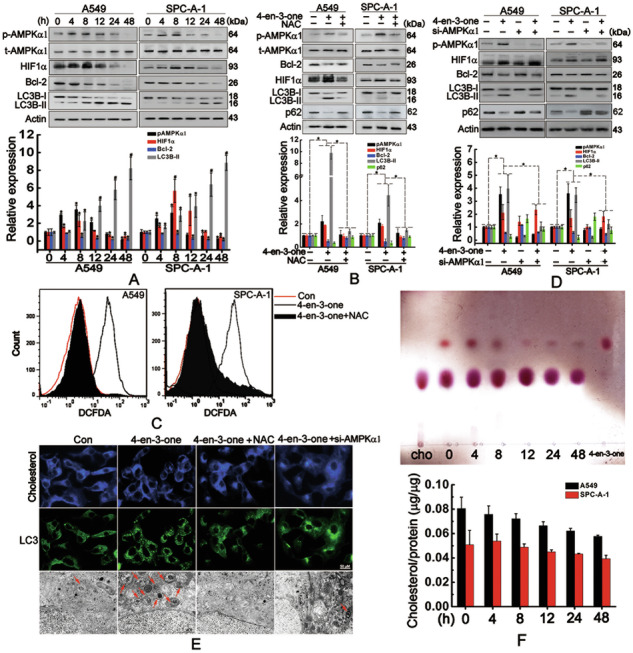


Amended Fig. 2
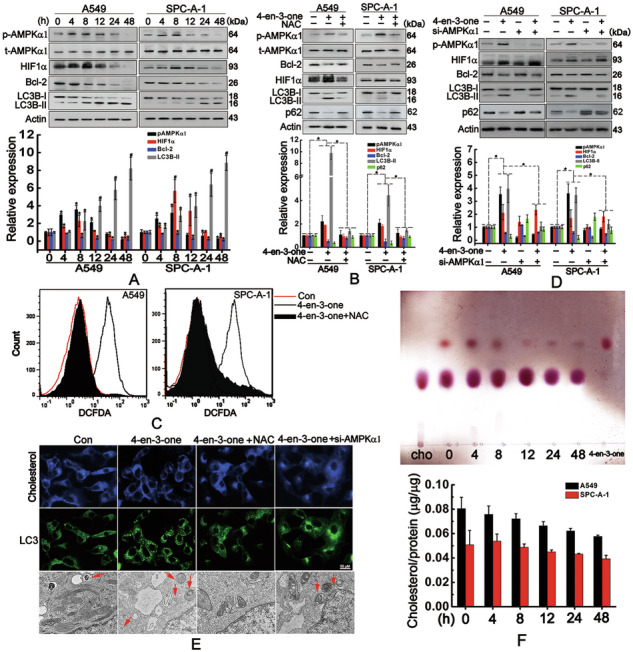


Original Fig. 3
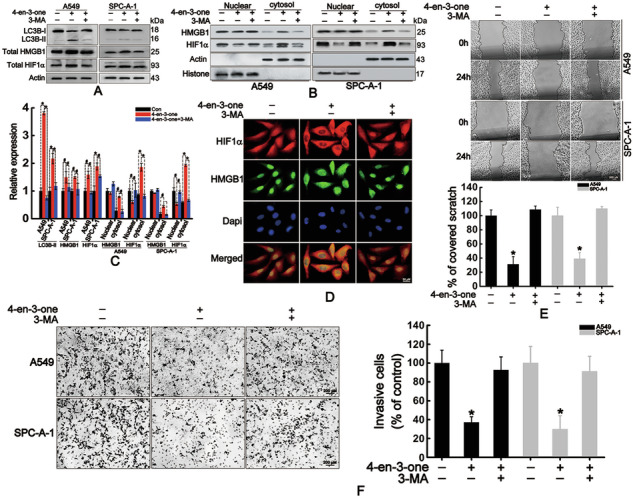


Amended Fig. 3