# Supplementary material for: 4-cholesten-3-one suppresses lung adenocarcinoma metastasis by regulating translocation of HMGB1, HIF1α and Caveolin-1
Source: Cell Death Dis. 2016 Sep 22;7(9):e2372–. doi: 10.1038/cddis.2016.281 (PMC5059879; doi:10.1038/cddis.2016.281)
Supplement: Supplementary Informations [file cddis2016281x1.doc]

Materials and Methods

HPLC analysis

Serum was obtained by centrifugation at 5000 rpm for 10 min, and stored at -80 ^o^C until analysis. Sterols were extracted by chloroform/methanol (2:1). After removal of solvents using nitrogen, the residues were dissolved in 90% methanol and applied to HPLC analysis with Venusil MP C18 column (4.6mm×250mm, Agela Technologies Inc). Methanol/water (90:10 v/v, containing 0.01% trifluoroacetic acid) served as the mobile phase at a flow rate of 0.8ml/min at 40^o^C. 4-cholesten-3-one was detected at 242nm and quantified according to the internal standard.

Table S1 The feed consumption of mice.

| **Group** | **Feed consumption （g/week/mouse）** | | | | | | | | |
| --- | --- | --- | --- | --- | --- | --- | --- | --- | --- |
|  | 1 | 2 | 3 | 4 | 5 | 6 | 7 | 8 | 9 |
| **Control diet** | 35.6±1.9 | 37.8  ±1.5 | 37.4  ±1.3 | 38.5  ±2.0 | 38.4  ±1.7 | 38.2  ±2.1 | 37.9  ±2.4 | 37.9  ±2.3 | 38.8  ±1.4 |
| **4-cholesten-3-one** | 36.9±2.1 | 38.7  ±1.7 | 39.1  ±2.3 | 39.2  ±1.8 | 39.3  ±1.9 | 38.4  ±1.6 | 38.9  ±1.2 | 39.3  ±1.9 | 38.7  ±2.0 |

Figure S1


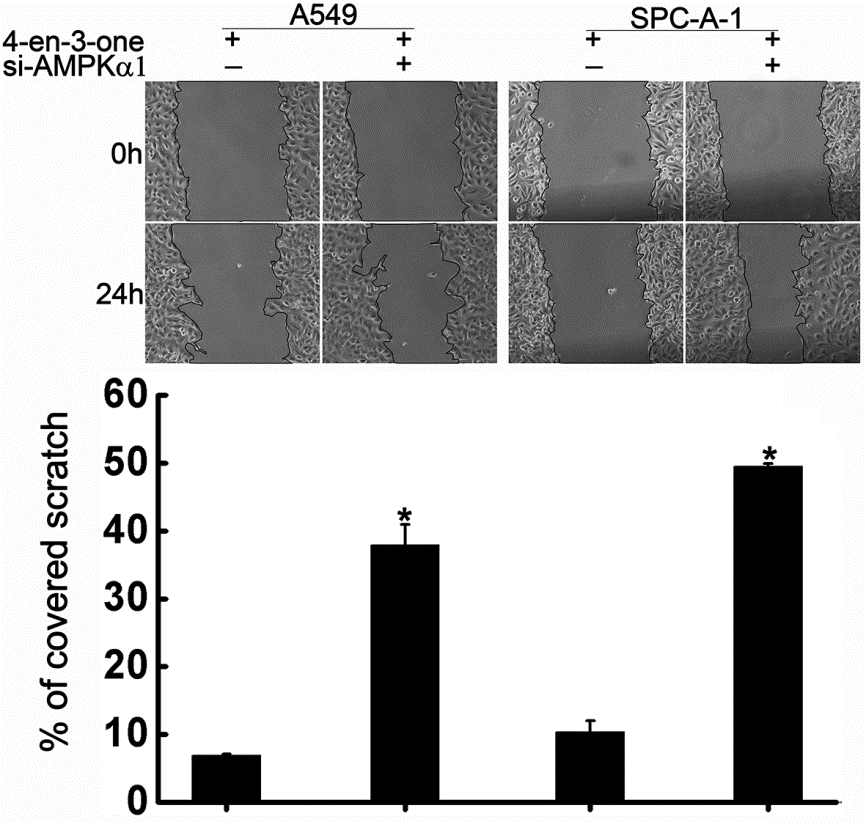


Fig.S1 ADC cells were transfected with siRNA against AMPKα1, starved for 4h, and then treated with 10 μM 4-en-3-one for 24h. ADC cells migration was determined by scratch assay. *P<0.01.

Figure S2


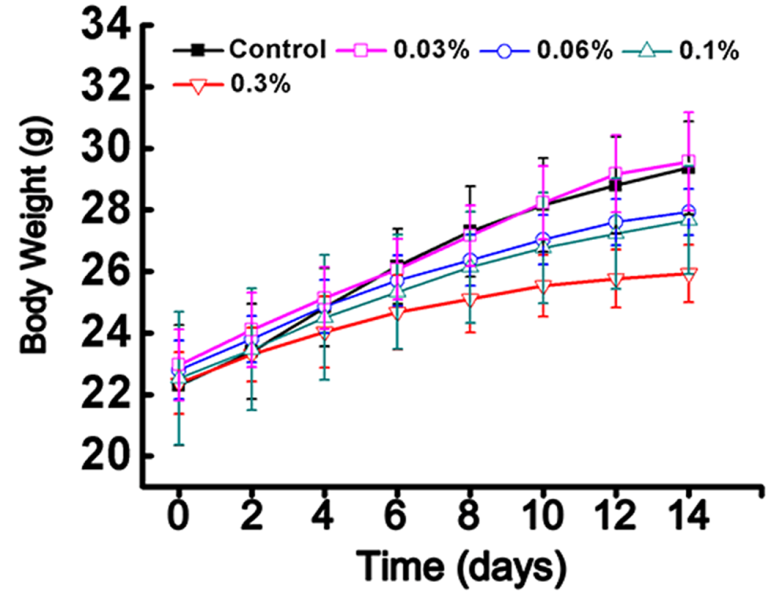


Fig.S2 Time curves of body weight of mice fed with different dose of 4-cholesten-3-one.

Figure S3


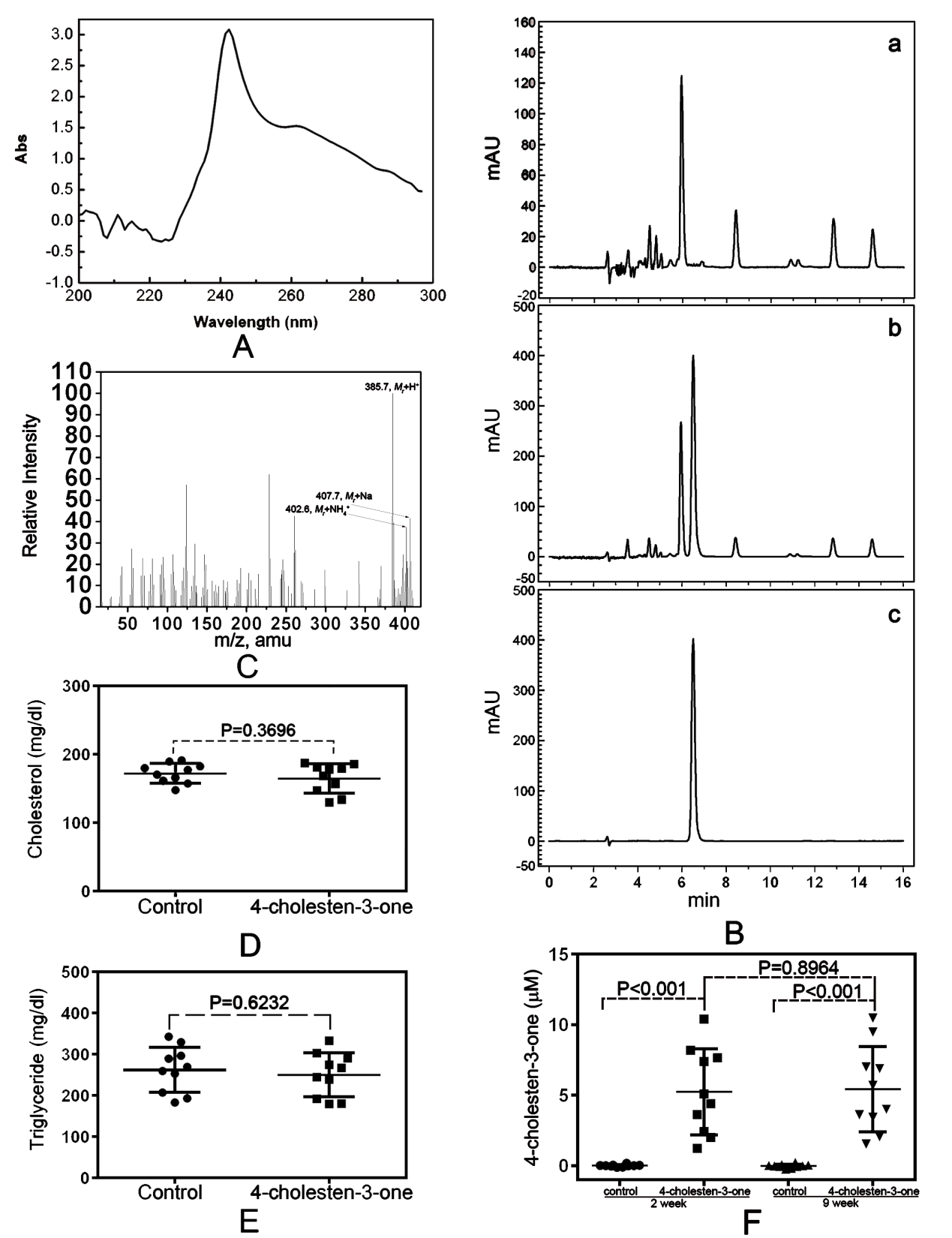


Fig.S3 Serum cholesterol, triglyceride and 4-cholesten-3-one of mice fed with control diets or 4-cholesten-3-one-supplemented diets. A. [Ultraviolet scanning](http://dict.cnki.net/dict_result.aspx?searchword=紫外扫描&tjType=sentence&style=&t=ultraviolet+scanning) of 4-cholesten-3-one. B. HPLC analysis of serums from mice fed with control diets or 4-cholesten-3-one-supplemented diets. a. HPLC analysis of serum from mice fed with control diets; b. HPLC analysis of serum from mice fed with 4-cholesten-3-one-supplemented diets; c. HPLC analysis of standard 4-cholesten-3-one. C. Mass spectrum analysis of the suspected 4-cholesten-3-one. *M_r_*+H, represented molecular weight of product plus H^+^; *M_r_*+Na, represented molecular weight of product plus Na^+^; *M_r_*+NH_4_^+^, represented molecular weight of product plus Na^+^. E. Serum cholesterol concentration of mice. F. Serum triglyceride concentration of mice. F. Serum 4-cholesten-3-one concentration of mice fed with control diets or 4-cholesten-3-one-supplemented diets for two weeks or nine weeks.
